# *Bursaphelenchus suri* n. sp.: A second *Bursaphelenchus* syconial parasite of figs supports adaptive radiation among section *Sycomorus* figs

**DOI:** 10.1371/journal.pone.0265339

**Published:** 2022-04-06

**Authors:** Natsumi Kanzaki, Meike S. Kruger, Jaco M. Greeff, Robin M. Giblin-Davis

**Affiliations:** 1 Kansai Research Centre, Forestry and Forest Products Research Institute, Momoyama, Fushimi, Kyoto, Japan; 2 Department of Biochemistry, Genetics and Microbiology, University of Pretoria, Pretoria, South Africa; 3 Department of Entomology and Nematology, Fort Lauderdale Research and Education Centre, University of Florida/IFAS, Davie, FL, United States of America; University of Limpopo, SOUTH AFRICA

## Abstract

The nematode genus *Bursaphelenchus* is a highly divergent group. This genus mainly consists of mycophagous entomophilic species, but some species have specialized as obligate or facultative plant parasites, facultative insect parasites, or exhibit feeding dimorphism (phenotypic plasticity) leading to mycophagous and predatory forms. In the present study, a new *Bursaphelenchus* species, *B*. *suri* n. sp. was isolated from fresh syconia (figs) of *Ficus sur* and is described and illustrated based on its typological characters and molecular phylogenetic status. The new species is characterized by its highly derived feeding structures found in obligate plant parasites, lip possessing a labial disc and a long and thick stylet with a long conus and extremely well-developed basal swellings. In addition, slender body of both sexes is characteristic of the species. The new species is phylogenetically and typologically closely related to *B*. *sycophilus*, i.e., these two species share the characteristic feeding structures and form a well-supported clade within the *B*. *fungivorus* group in the genus. Biologically, these two species are both isolated from fresh figs of the section *Sycomorus*. However, the new species differs from *B*. *sycophilus* by the length of the female post-uterine sac and the shape of the male spicule, i.e., the new species has a long post-uterine sac and spicule condylus without dorsal recurvature. Thus, the new species is the second obligate fig parasite of the genus, and the evolutionary relationship between the *B*. *suri* n. sp. and *B*. *sycophilus* clade and section *Sycomorus* figs is hypothesized as an example of adaptive radiation with more species to be discovered.

## Introduction

The specific pollination system of figs (*Ficus* spp.) and fig wasps has been studied as a model system for co-evolution and diversification [1–4]. There, several other invertebrates, e.g., mites and nematodes are involved as phoretic associates and parasites of the wasps, plant (syconia tissue) parasites, microbe feeders and predators [5–9]. For example, the nematode genus *Parasitodiplogaster* Poinar has been examined as a case study of host-parasite virulence evolution [5] and *Pristionchus* Kries was revealed as a radiation of nematodes that manifest extreme trophic diversity through divergent developmental phenotypes in section *Sycomorus* figs [10]. After the early reports of fig/fig wasp-associations with nematodes [11, 12], more than ten genera of nematodes have been reported to have specialized associations with the fig pollination system [9–11, 13–19].

The nematode superfamily Aphelenchoidoidea is a divergent group in terms of feeding life history traits and insect (invertebrate) associations. The superfamily is derived from soil-dwelling fungal feeders, and currently contains fungal feeders, plant parasites, insect parasites, and predators [20–22]. Within this superfamily, there are at least four obligate fig/fig wasp associated lineages, where the nematodes are carried by fig wasps, and feed on internal syconia tissues of figs [17, 19]. In addition, all four lineages are phylogenetically separable from each other and are sisters to corresponding mycophagous lineages [17, 19], suggesting that these four clades occurred independently from mycophagous lineages. Interestingly, the ingestive (stomatal) structure of all four lineages are similar to each other in being specialized for plant-parasitism, i.e., a morphological and functional convergence has occurred [17, 19], and these lineages could be an exciting system to study adaptive radiation and the origins of nematode plant parasitism.

In previous studies, Kanzaki et al. [17] and Kruger et al. [23] reported that two species of the genus *Bursaphelenchus* Fuchs are associated with figs, i.e., *B*. *sycophilus* from *F*. *variegata* (Blume) in Japan [17] and an undescribed *Bursaphelenchus* sp. from *F*. *sur* Forssk. in South Africa [23]. The purpose of this study was to describe and illustrate *Bursaphelenchus suri* n. sp. to elucidate its close relationship with *B*. *sycophilus* from Japan, which it shares specialized morphology and biology within *Sycomorus* figs.

## Materials and methods

### Ethics statement

Specific permissions were not required for the nematodes collected for the present study. The fields (trees) used for nematode collection were on the grounds of the University of Pretoria campus. Endangered or protected species were not involved with the present study.

### Nematode collection

The procedures for collecting specimens were provided in the previous study [23]. In short, various-phased syconia of *F*. *sur* were collected from trees planted on the grounds of the campus of the University of Pretoria, South Africa (GPS: 25°45’20” S, 28°13’40”E, 1370 m a.s.l.) in Nov. 2015, cut into small pieces in distilled water, and nematodes were hand-picked from the water with a stainless-steel insect pin under a dissecting microscope.

Collected nematodes were morphologically studied for genus or family-level identification. Some specimens were heat-killed at 55°C for one min. and fixed in TAF (2.0% triethanolamine, 7.0% formalin, 91% distilled water) for morphological vouchers, while the others were fixed in DESS [24] for molecular profiles.

### Morphological observation

The TAF-fixed materials were processed to glycerin and mounted according to the modified Seinhorst method [25] and the Maeseneer and d’Herde method [26], respectively. The mounted materials were observed under a light microscope (Eclipse 80i, Nikon). Morphological drawings and morphometric analyses were conducted using a drawing tube attached to the microscope. Photomicrographs were taken using a digital camera system (MC170 HD, Leica). All drawings and micrographs were edited to construct figures using Photoshop 2020 (Adobe).

### Molecular profiles and phylogeny

The molecular sequences of ca 1.7 kb of the small subunit (SSU) and ca 0.7 kb of the D2-D3 expansion segments of the large subunit (D2-D3 LSU) ribosomal RNA genes and ca 0.6 kb of mitochondrial cytochrome oxidase subunit I (mtDNA) were determined and deposited to the GenBank database in the previous study [23]. Briefly, DESS-fixed nematodes were rehydrated, observed under the microscope for typological identification, and individually transferred to nematode digestion buffer [27, 28]. The nematodes were digested at 55°C for 30 min. and used for PCR template to determine the sequences according to the methodologies of Ye et al. [29] and Kanzaki and Futai [30].

For the phylogenetic analysis, compared sequences were selected according to the results of a BLAST homology search and previous studies [31, 32], i.e., because the BLAST search suggested that the new species of nematode was closest to *B*. *sycophilus* Kanzaki, Tanaka, Giblin-Davis & Davies, and belonged to the *fungivorus* group of the genus, those nematode species belonging to the *fungivorus* group and several other *Bursaphelenchus*, *Parasitaphelenchus* Fuchs, *Ruehmaphelenchus* Goodey and *Sheraphelenchus* Nickle species belonging to other parasitaphelenchid clades were used for the Bayesian phylogenetic analyses based on SSU and D2-D3 LSU.

The compared sequences were aligned separately using MAFFT (available online at http://mafft.cbrc.jp/alignment/server/) [33, 34], and the base substitution model was determined using MEGA7 [35] under the Akaike information criterion (AIC) for model selection. Bayesian analysis was performed separately to confirm the tree topology of each gene using MrBayes 3.2 [36, 37]; four chains were run for 4 × 10^6^ generations. Markov chains were sampled at intervals of 100 generations [38]. Two independent runs were performed, and, after confirming the convergence of runs and discarding the first 2 × 10^6^ generations as burn-in, the remaining topologies were used to generate a 50% majority-rule consensus tree.

### Nomenclatural acts

The electronic edition of this article conforms to the requirements of the amended International Code of Zoological Nomenclature, and hence the new names contained herein are available under that Code from the electronic edition of this article. This published work and the nomenclatural acts it contains have been registered in ZooBank, the online registration system for the ICZN. The ZooBank LSIDs (Life Science Identifiers) can be resolved and the associated information viewed through any standard web browser by appending the LSID to the prefix "http://zoobank.org/". The LSID for this publication is: urn:lsid:zoobank.org:pub:011FAF51-3B7C-4D4C-9228-CF0DC12B164D. The electronic edition of this work was published in a journal with an ISSN, and has been archived and is available from the following digital repositories: PubMed Central and LOCKSS.

## Results

### Phylogenetic status

**Fig 1:**
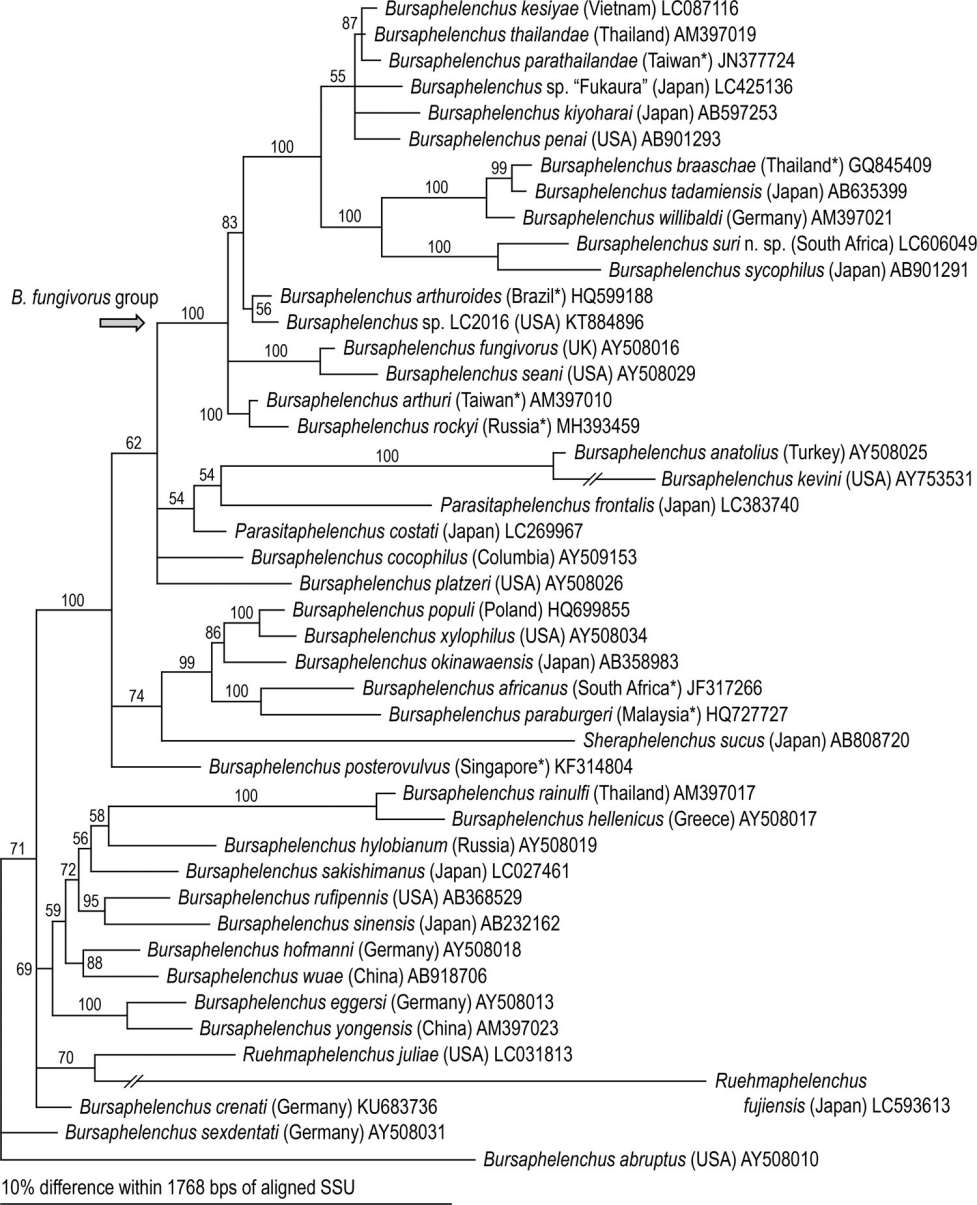
Bayesian tree inferred from SSU under GTR+G+I model. For model selection, AIC = 19854.013; lnL = -9829.881. Analytical parameters are: freqA = 0.26, freqC = 0.19, freqG = 0.27, freqT = 0.28; R(a) = 1.11, R(b) = 2.70, R(c) = 1.46, R(d) = 0.54, R(e) = 4.66, R(f) = 1.00; Pinva = 0.35; Shape = 0.31. Posterior probability values exceeding 50% are given on appropriate clades. The type locality of each species marked with an asterisk suggests that the material was isolated from wooden packing material imported from that country.

**Fig 2:**
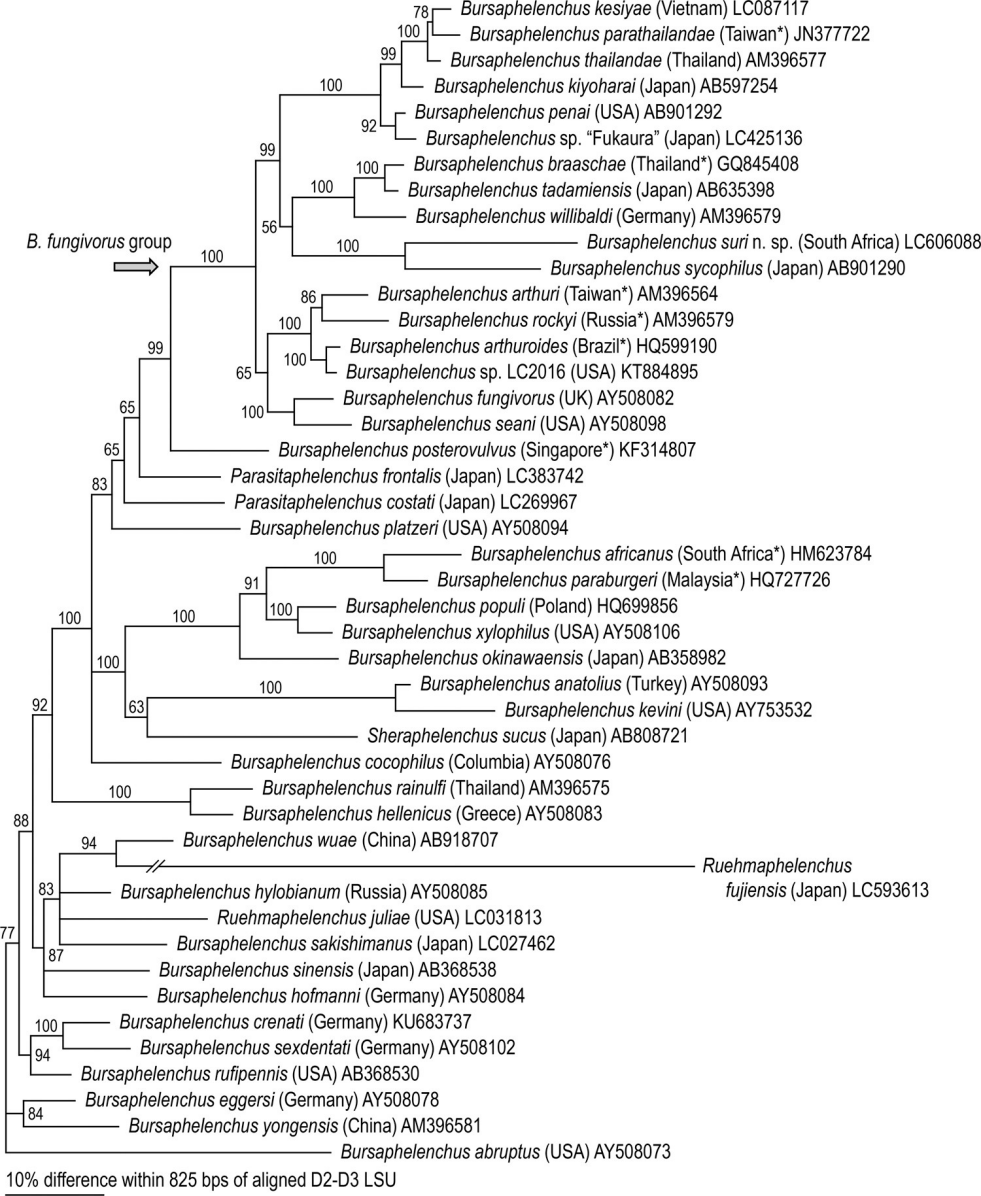
Bayesian tree inferred from D2-D3 LSU under GTR+G+I model. For model selection, AIC = 24371.263; lnL = -12088.342. Analytical parameters are: freqA = 0.21, freqC = 0.19, freqG = 0.32, freqT = 0.28; R(a) = 0.17, R(b) = 1.68, R(c) = 0.53, R(d) = 0.34, R(e) = 3.05, R(f) = 1.00; Pinva = 0.32; Shape = 0.88. Posterior probability values exceeding 50% are given on appropriate clades. The type locality of each species marked with an asterisk suggests that the material was isolated from wooden packing material imported from that country.

*Bursaphelenchus suri* n. sp. belongs to subgroup 1 of the *fungivorus* group sensu Kanzaki et al. [31], and is phylogenetically closest to *B*. *sycophilus*, the other fig-associated species of the genus (Figs 1 and 2). These two species formed a maximally supported clade within the group.

### Taxonomic description

**Fig 3:**
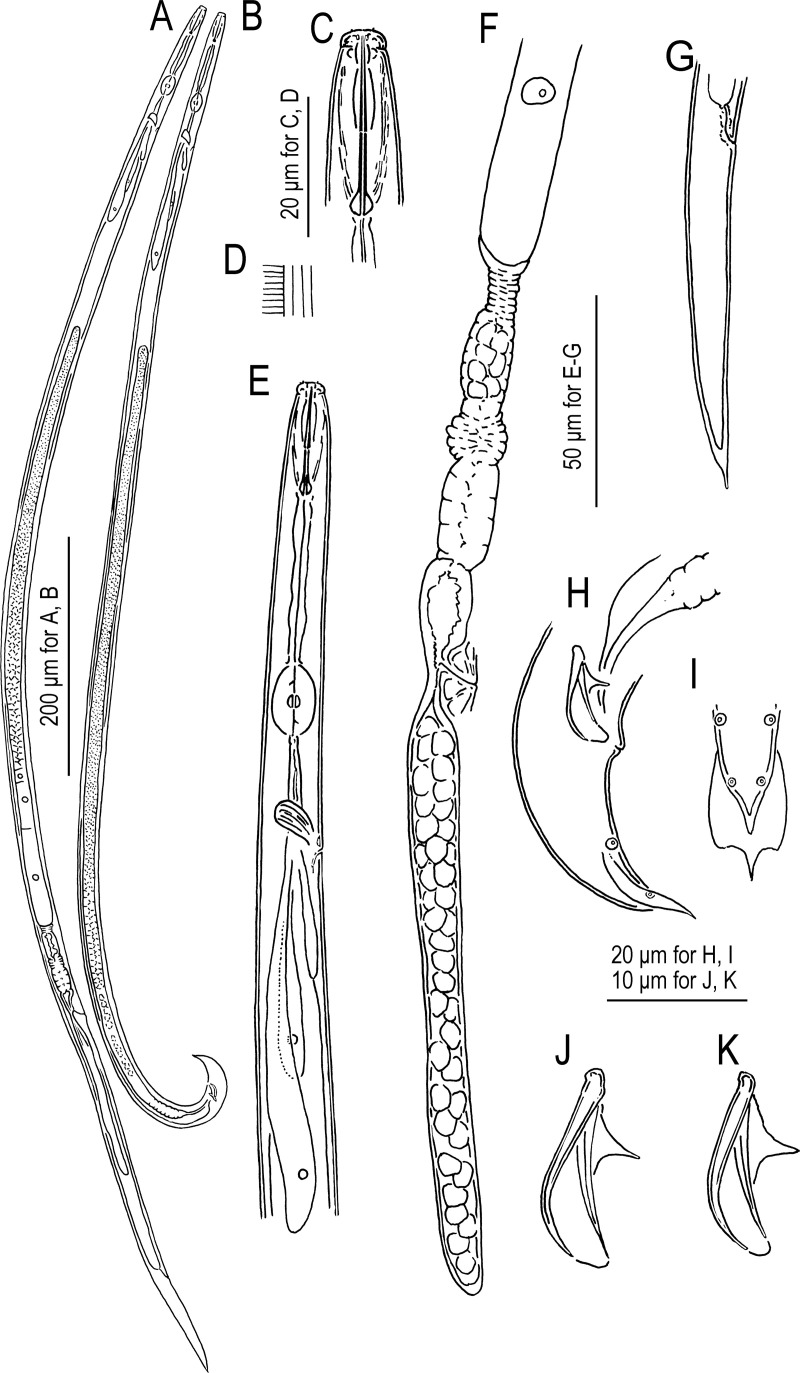
Morphological drawings for adult *Bursaphelenchus suri* n. sp. A: Female; B: Male; C: Lip and stylet region of male; D: Body surface of male showing lateral field and annulations; E: Head to pharyngeal region of male; F: Posterior part of female gonad; G: Female tail; H: Male tail; I: Male tail tip; J, K: Male spicule. All subfigures are in the right lateral view except for H (ventral view).

**Fig 4:**
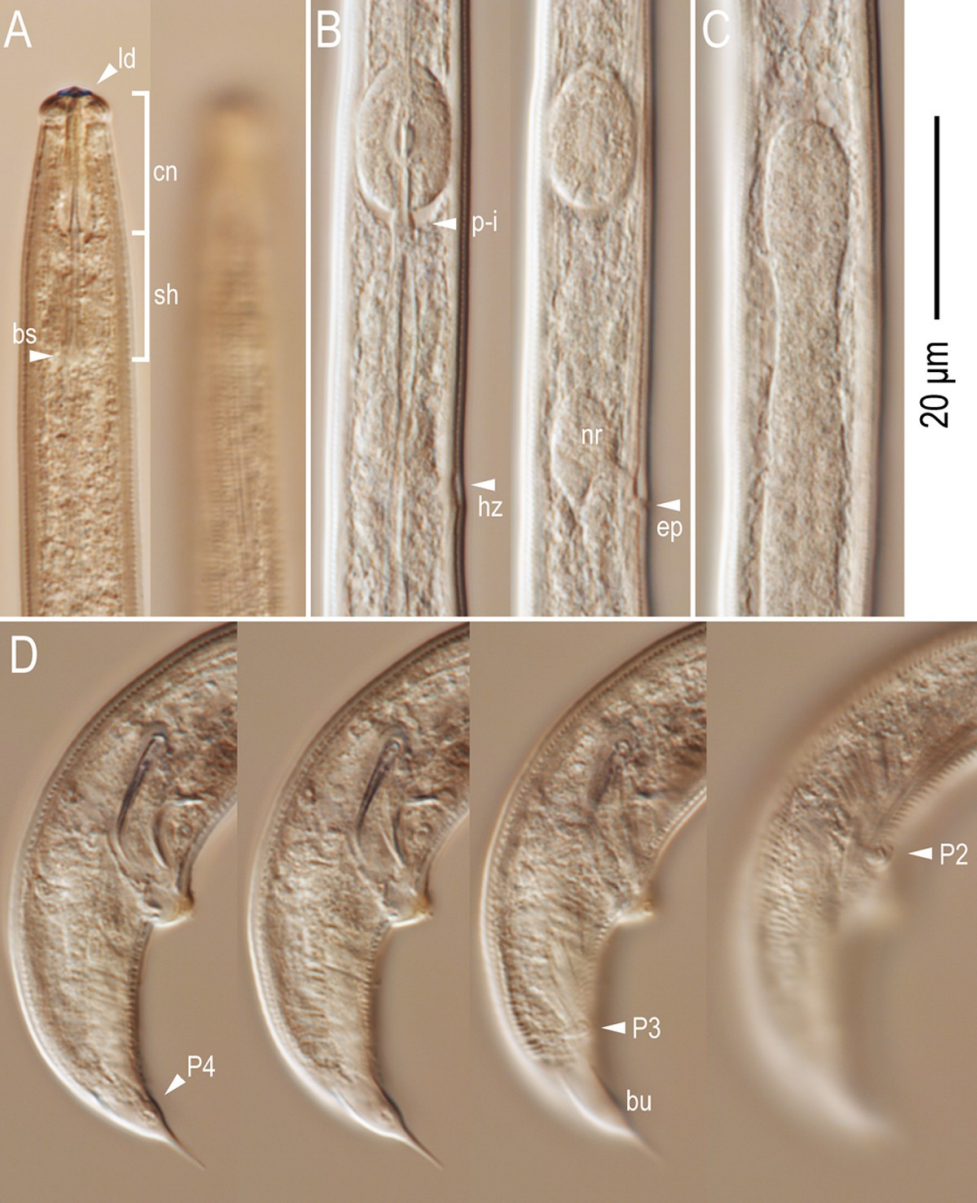
Light micrographs for male *Bursaphelenchus suri* n. sp. A: Lip and stylet region in two different focal planes (ld = labial disc; cn = stylet conus; sh = stylet shaft; bs = basal swelling); B: Pharyngeal part in two different focal planes (p-i = pharyngo-intestinal junction; hz = hemizonid; nr = nerve ring; ep = secretory-excretory pore); C: Anterior end of testes; D: Male tail in four different focal planes (P + number = genital papillae; bu = bursal flap). All subfigures are in right lateral view.

**Fig 5:**
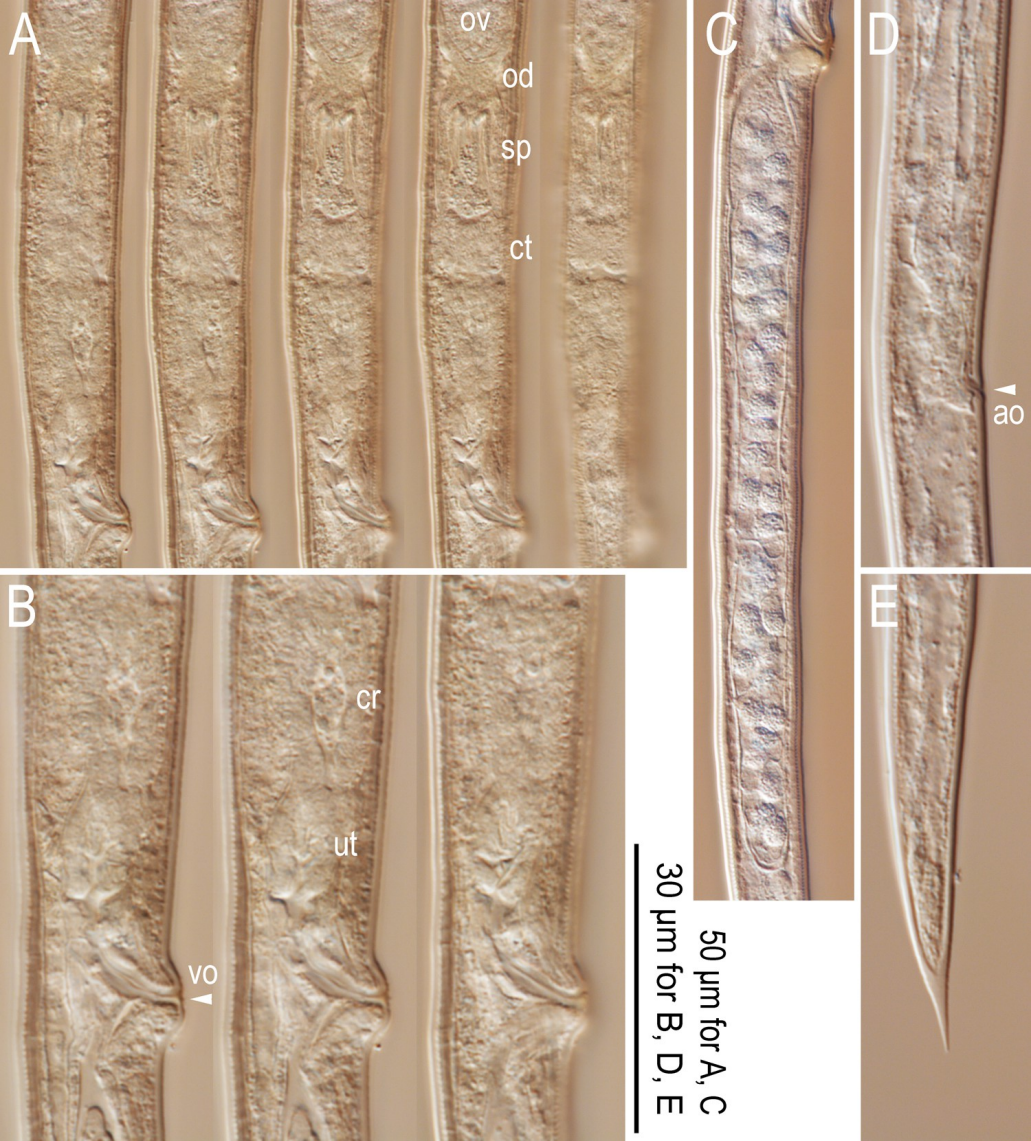
Light micrographs for female *Bursaphelenchus suri* n. sp. A: Ovary to vulval region in five different focal planes (ov = ovary; od = oviduct; sp = spermatheca; ct = connective tissue); B: Vulval region in three different focal planes (vo = vulval opening; cr = crustaformeria; ut = uterus); C: Post-uterine sac; D: Anal region (ao = anal opening); E: Tail tip. All subfigures are in right lateral view.

*Bursaphelenchus suri* Kanzaki, Kruger, Greeff & Giblin-Davis n. sp. urn:lsid:zoobank.org:act:7A4B50E1-451A-4A73-9034-0106BD144756 (Figs 3–5).

#### Description

According to the journal’s requirement, a short description of the comparative diagnostic characters is given here. The typical morphologies for the genus and species group appear in the short description are provided in the previous species descriptions [16, 31, 32]. A detailed typological description is given in S1 Text.

*Adult*. Relatively large and slender species of the genus. Body shape and cuticle structures are typical to the genus, with four-lined lateral field, but the structure is vague, and internal lines are difficult to observe. Lip with six equal-sized sectors, roundish rectangular to triangular in lateral view. A labial disc present at the anterior end, and its edge appears as two short projections in lateral view. Stylet with narrow lumen separated into a long conus and a shaft with clear, very well-developed and somewhat tear-drop-shaped basal swellings. Procorpus with clear procorpal tube, about two stylet lengths long, metacorpus (= median bulb) well-developed, and pharyngo-intestinal junction structures are typical to the genus. Dorsal pharyngeal gland *ca* 7–10 metacorpal lengths long. Nerve ring at about one stylet length (about 1.5 metacorpal lengths) posterior to metacorpus. Hemizonid about 1.5 stylet length (about 2 metacorpal lengths) posterior to metacorpus. Secretory-excretory pore at immediately posterior or almost same level of hemizonid.

*Male*. Body and gonadal structure are typical to the genus. Anterior end of testis outstretched (8 out of 9 type specimens) or reflexed (1 out of 9). Spermatocytes arranged in multiple (3–5) rows. Spicules typical for the *fungivorus* group of the genus. Gubernaculum absent. Tail smoothly tapering in anterior 2/3, and distal 1/3 narrowing abruptly. Bursal flap conspicuous, an oval shape with a triangular projection at the posterior end. Three pairs of genital papillae present, but ventral precloacal papilla (P1) which is present in most other parasitaphelenchid nematodes not observed in light microscope, possibly vestigial.

*Female*. Structure and position of reproductive tract typical to the genus. A pair of three-celled structures usually found in the genus was not confirmed, possibly because of material condition, but this region was somewhat sclerotized. Vulva without any flap apparatus. Post-uterine sac long, 6.3–9.7 vulval body diam. long, extending for almost half or more (47–73%) of vulva to anus distance. Tail slender, 5.6–10.7 anal body diam. long, elongate conoid in shape, smoothly tapering to pointed terminus.

#### Morphometrics

Morphometric values are summarized in Table 1.

**Table 1:** Morphometrics of type specimens of *Bursaphelenchus suri* n. sp. All measurements are in μm and in the form: mean ± standard deviation (range).

	Male		Female
	Holotype	Paratypes	Paratypes
n	1	8	10
L	1030	969 ± 95 (833–1096)	1211 ± 177 (950–1629)
a	60.0	56.5 ± 4.6 (50.5–61.8)	59.2 ± 6.1 (51.1–69.1)
b	11.9	11.6 ± 1.0 (10.1–12.9)	14.8 ± 2.4 (11.5–20.7)
c	29.6	28.0 ± 1.8 (25.4–31.4)	13.3 ± 0.8 (11.8–14.7)
c’	2.1	2.2 ± 0.2 (1.8–2.6)	7.5 ± 1.4 (5.6–10.7)
T or V	72.4	69.4 ± 2.6 (67–73)	72.7 ± 1.7 (70–75)
M	52.0	54.0 ± 2.7 (50.0–58.6)	53.5 ± 1.7 (51.4–56.8)
Maximum body diam.	17.2	17.2 ± 1.8 (15.2–21.7)	20.5 ± 2.2 (17.9–25.0)
Lip diam.	6.8	6.7 ± 0.2 (6.4–7.1)	6.9 ± 0.3 (6.4–7.1)
Lip height	3.6	3.3 ± 0.4 (2.9–3.6)	3.5 ± 0.4 (2.9–4.3)
Lip height/diam.	1.9	2.1 ± 0.2 (1.9–2.4)	2.0 ± 0.2 (1.7–2.3)
Stylet conus	13.0	13.6 ± 0.8 (12.9–15.0)	13.6 ± 1.0 (11.4–15.0)
Stylet length	25.0	25.2 ± 1.4 (22.9–27.1)	25.4 ± 1.5 (21.4–26.4)
Metacorpus diam.	9.3	9.6 ± 0.5 (9.3–10.7)	10.5 ± 0.6 (10.0–11.8)
Metacorpus length	15.7	16.2 ± 0.9 (15.0–17.9)	16.8 ± 0.8 (15.7–18.6)
Metacorpus length/diam. ratio	1.7	1.7 ± 0.1 (1.5–1.9)	1.6 ± 0.1 (1.4–1.9)
Nerve ring from anterior end	108	104 ± 3.4 (100–108)	102 ± 4.2 (94–109)
Relative position of nerve ring ^a^	1.4	1.3 ± 0.2 (1.0–1.5)	1.2 ± 0.2 (1.0–1.5)
Hemizonid from anterior end	113	109 ± 4.4 (103–114)	112 ± 5.7 (100–119)
Relative position of hemizonid^a^	1.7	1.6 ± 0.3 (1.1–2.0)	1.8 ± 0.3 (1.1–2.3)
Secretory-excretory pore from anterior end	114	111 ± 4.6 (104–116)	113 ± 5.6 (101–120)
Relative position of secretory-excretory pore^a^	1.8	1.7 ± 0.3 (1.2–2.1)	1.8 ± 0.3 (1.1–2.4)
Testis or ovary length	746	673 ± 81 (557–779)	613 ± 69 (457–696)
Length of reflexed part of gonad	0	41 (n = 1)	0
Cloacal or anal body diam.	16.7	16.1 ± 1.1 (14.6–18.2)	12.2 ± 0.7 (11.4–13.2)
Tail length	35	35 ± 2.2 (32–39)	92 ± 18 (66–138)
Spicule (chord)	14.6	14.9 ± 0.6 (14.1–15.7)	-
Spicule (curved along dorsal limb)	17.6	17.2 ± 0.4 (16.7–17.7)	-
Vulval body diam.	-	-	19.1 ± 1.6 (17.1–23.2)
Vulva-anus distance	-	-	240 ± 44 (178–330)
Post-uterine sac (PUS) length	-	-	142 ± 18 (124–187)
PUS % to vulva-anus distance	-	-	60.0 ± 8.1 (47.2–73.4)
PUS / vulval body diam.	-	-	± 1.0 (6.3–9.7)

^a^ Calculated with a formula: Distance from posterior end of metacorpus to each organ (nerve ring, hemizonid, or secretory-excretory pore) / metacorpal length.

#### Type material

Holotype male (Collection accession number: 51322), eight paratype males (51323–51330), and ten paratype females (51331–51340) deposited in the National Collection of Nematodes (NCN) at the Nematology Unit, Biosystematics, ARC-Plant Health and Protection, Roodeplaat, Pretoria.

#### Type habitat and locality

The type material was obtained from a syconium of *Ficus sur* growing on the campus of the University of Pretoria, Pretoria, South Africa (GPS: 25°45’20” S, 28°13’40”E, 1370 m a.s.l.) in November 2015.

#### Diagnosis and relationship

In addition to generic characters, *Bursaphelenchus suri* n. sp. is characterized by its lip structure, possessing a labial disc; long stylet with long conus occupying more than half of the total stylet length and large basal swellings; four-lined inconspicuous lateral field, male spicule with slightly dorsally truncate and roundish-squared condylus, dorsal and ventral limbs and membrane-like part between the limbs; bursal flap with a conspicuous projection; female gonad without clearly branching spermatheca; and relatively long female tail.

Based on its lip and stylet morphology and biology, i.e., association with a section *Sycomorus* fig, *B*. *suri* n. sp. is obviously close to *B*. *sycophilus*, i.e., forming a well-supported clade with the other fig-syconia-parasite. The phylogenetic status within the group corroborates their typological and biological similarities (Figs 1–5) [17, 23]. However, the new species can be distinguished from *B*. *sycophilus* by typological differences in adult morphology, namely the post-uterine sac length in females (6–10 times vulval body diameter, extending for almost half or more (47–73%) of the vulva to anus distance in *B*. *suri* n. sp. vs 3–5 times vulval body diameter, extending less than half (40–48%) of the vulva to anus distance in *B*. *sycophilus*) and condylus shape in male spicules (slightly dorsally truncate and roundish-squared in *B*. *suri* n. sp. vs strongly dorsally arcuate in *B*. *sycophilus*).

*Bursaphelenchus maxbassiensis* (Massey) Baujard also shares a modification of lip structure and stylet with long conus and large basal swellings with the two fig-parasites [16, 17, 39, 40]. However, *B*. *suri* n. sp. is distinguished from *B*. *maxbassiensis* by its lip shape, roundish square in lateral view with a labial disc vs laterally expanded to form an umbrella-like shape, and the labial disc was not observed; position of secretory-excretory pore, posterior vs anterior to metacorpus; male spicule morphology, with slightly dorsally truncate and roundish-squared condylus and relatively clear dorsal and ventral limbs vs dorsally recurved and pointed condylus and without clear ventral limb; male bursal flap shape, with pointed vs rounded tip; female tail shape long vs short conoid [16, 39].

## Discussion

### Biological characters of the *B*. *fungivorus* group

The new species and its closest relative, *B*. *sycophilus* belong to subgroup 1 of the *fungivorus* group of the genus [31]. Although most *Bursaphelenchus* spp. are generally associated with wood-boring beetles, particularly bark beetles that inhabit the above ground parts of dead trees [20, 41–43], the *fungivorus* group contains several soil-inhabiting species (*B*. *hunti* (Steiner) Giblin & Kaya, *B*. *fungivorus* Franklin & Hooper, *B*. *gonzalezi* Loof, *B*. *seani* Giblin & Kaya and *B*. *rockyi* Wang, Fang, Maria, Gu & Ge) [44–48], and their insect associations are rather variable compared with other intrageneric groups, e.g., soil-dwelling bees (*B*. *seani*) [47], a stag beetle (*B*. *tadamiensis* Kanzaki, Taki, Masuya & Okabe) [49], ambrosia beetles (*B*. *kiyoharai* Kanzaki, Maehara, Aikawa, Masuya & Giblin-Davis and *B*. *penai* Kanzaki, Giblin-Davis, Carrillo, Duncan & Gonzalez) [50, 51], and fig wasps (*B*. *sycophilus* and *B*. *suri* n. sp.) [17, 23]. In addition to the loss of the bursal flap in *B*. *kiyoharai* and *B*. *penai*, *B*. *kiyoharai*, *B*. *penai*, *B*. *sycophilus* and *B*. *suri* n. sp. have also lost (or possess vestigial) a P1 genital papilla in males [50, 51]. Considering the central phylogenetic placement within the genus [42, 43], the loss of a bursal flap and P1 papilla among typical *Bursaphelenchus* species (with a plesiomorphic P1 papilla and bursal flap) supports some genetic plasticity in the genus and the group.

Phylogenetically, the two fig-associates were sister to a clade containing *B*. *braaschae* Gu & Wang, *B*. *tadamiensis* and *B*. *willibaldi* Schönfeld, Braasch & Burgermeister (Fig 1). Within these three species, *B*. *tadamiensis* has been isolated from a stag beetle collected in Fukushima, Japan, i.e., the species inhabits the decaying wood of a broad-leaved tree [49], but the biological characters of the other two species are unknown because they were isolated from wood packing materials [52, 53]. Currently, we do not have a clear understanding of the evolutionary history of fig-associated *Bursaphelenchus*, e.g., how their niche and feeding preferences evolved. Although it seems likely that it involved an early introduction of a *fungivorus* group shared ancestor into a sycone of an ancestor fig of section *Sycomorus* by another insect vector with a host switch to fig wasps and a subsequent adaptive radiation involving the evolution from mycophagy to plant-parasitism. The genetic distances within the clade of fig-associates (*fungivorus* subgroup 1) and the nearest relatives of *fungivorus* subgroups 2 and 3 have relatively long branch lengths (Fig 1). This is somewhat reminiscent of another clade currently placed within a large *Bursaphelenchus* clade based upon molecular phylogenetic analysis. *Ruehmaphelenchus* appears to be associated and radiating chiefly with ambrosia beetles [54]. Thus, further survey work is justified for the 14 species of figs in the subsection *Sycomorus* and six species of the subsection *Neomorphe* in the subgenus and section of *Sycomorus* [55] (https://www.figweb.org/Ficus/Classification_of_figs/index.htm) which could reveal more new species and a clearer pattern of the evolutionary history and adaptive radiation of the fig-associated members of subgroup 1 of the *fungivorus* group. This was foreshadowed by Susoy et al. [10] in their paper reporting on the section *Sycomorus* radiation of at least seven species of *Pristionchus* where they also recovered *Bursaphelenchus* spp. from *F*. *sycomorus* L. and *F*. *mauritiana* Lam., but not *F*. *racemosa* L. (see Suppl. Table 3 in Susoy et al., [10]). In addition, during the sequencing of dauers from the pollinating fig wasp *Ceratosolen coecus* (Coquerel) emerging from ripe figs of *Ficus mauritiana*, no *Bursaphelenchus* sp. were recovered questioning if the fig wasp pollinator is the insect carrier of the *fungivorus* group radiation [10]. Kanzaki et al. [17] hypothesized that because the host-specific fig wasp, *Ceratosolen appendiculatus* (Mayr) was the only insect found in the syconium of *F*. *variegata* from which type materials of *B*. *sycophilus* were obtained, it was the likely carrier (or host) insect of the nematode. Using this logic, the likely carrier host for *B*. *suri* n. sp. would be *Ceratosolen capensis* Grandi since it is the primary pollinator of *F*. *sur* in South Africa, but fig wasps were not collected from *B*. *suri* n. sp. positive figs to sequence dauers for confirmation of the carrier association in the current study. Additionally, Kanzaki et al. [31] included *B*. *maxbassiensis* as a member of subgroup 1 of the *fungivorus* group because of similarities of its stylet morphology to *B*. *sycophilus*, but without molecular confirmation, this bark beetle associate may represent an example of convergence, which begs for re-isolation, sequencing, and further study. Further efforts to collect close relatives of fig-associated *Bursaphelenchus* followed by detailed biological and genetic analyses will help elucidate the evolutionary history of these highly derived species.

#### Some morphological characteristics of the new species and *B*. *sycophilus*

In addition to its fig association, *B*. *suri* n. sp. shares several important characters with *B*. *sycophilus*. Particularly, the presence of a labial disc, long conus and extremely well-developed basal swellings of the stylet, and slender body are highly characteristic.

Within the superfamily Aphelenchoidoidea, the labial disc has been confirmed in several *Ruehmaphelenchus* and *Aphelenchoides* Fischer species (fungal feeders) [56, 57], *Anomyctus xenurus* Allen (hypothesized to be a predator) [58, 59], and some species of the fig-associated genera, *Schistonchus* Cobb, *Ficophagus* Davies & Bartholomaeus and *Martininema* Davies & Bartholomaeus [19], but not in regular fungal feeders [20, 22]. The lip structure and stylet morphology are tightly related to their feeding habits, and the structures are hypothesized to be adapted to plant parasitism, where the nematodes penetrate host epidermal cells, which are physically harder than fungal hyphae. However, because the fig-associated species have not been cultured successfully, the usage of their lips and stylets have not been observed. Further culturing attempts are necessary for these species and also for the confirmation of plant parasitism within *Ruehmaphelenchus* spp., which has a typical fungal feeders’ stylet.

The slender body may be an adaptation for *B*. *suri* n. sp. and *B*. *sycophilus* to their habitat inside section *Sycomorus* figs. In the family Diplogastridae, which also contains several fig-associated lineages, several aquatic lineages, e.g., the aquatic group of *Allodiplogaster* Paramonov & Sobolev, have slender bodies [60]. Interestingly, *Teratodiplogaster* Kanzaki, Giblin-Davis, Davies, Ye, Center & Thomas and the fig-associated clade of *Pristionchus* sharing *Sycomorus* figs with *B*. *sycophilus* and *B*. *suri* n. sp. also have long and slender bodies, while most of the other non-*Sycomorus* fig-associates e.g., *Parasitodiplogaster* and *Caenorhabditis* Osche have rather stout bodies [61–63]. Considering their host/habitat fig species, the figs of section *Sycomorus* are filled with liquid, while others are not [55]. In fact, these lineages are crawling (swimming) in the cavity of figs filled with liquid during the interfloral phase [10, 62]. Similar to the tendency in Diplogastridae, where swimmers have long and slender bodies, *B*. *suri* n. sp. and *B*. *sycophilus* are considered to inhabit the cavity of the figs filled by the liquid, feeding on the internal surface tissue of figs. Contrastingly, the *Ficophagus* sp. sharing the same fig with *B*. *suri* n. sp. has a stouter body [23], and it is assumed to be a more sedentary species inhabiting the fig tissue. Thus, although both species are plant parasites and feed on fig tissue, their niches may be segregated within the fig.

#### Remarks on future studies

The section *Sycomorus* fig-associated *Pristionchus* spp. manifest feeding polymorphism to occupy a variety of niches according to the age of the figs [10]. Examination of the syconial ecology of section *Sycomorus* figs with different nematode faunal components could help elucidate how different species utilize and compete for different temporal and spatial resources. Such specialization could explain how the clade-specific radiations of certain nematode groups, such as *Pristionchus*, *Teratodiplogaster*, and *Bursaphelenchus* was triggered.

The tripartite relationship among fig, fig wasp, and nematodes is an intriguing system for the study of evolutionary biology. In addition, microbes and other invertebrates, e.g., mites, are involved in the system. However, most studies have been conducted as field surveys because of difficulties in the establishment of cultured materials for experimental research. To examine the system in more detail, laboratory strains will be a big advance. Of the animal interactants, nematodes are most predisposed to being cultured in the laboratory, e.g., *Caenorhabditis inopinata* can be cultured, and utilized as a satellite model system [9]. Further attempts at culturing fig-associated nematodes, including *Bursaphelenchus* species, should be undertaken to allow for more extensive studies.

## Supporting information

S1 Text Typological description of *Bursaphelenchus suri* n. sp. in traditional telegraphic style. (DOCX)
