# Supplementary material for: Bursaphelenchus suri n. sp.: A second Bursaphelenchus syconial parasite of figs supports adaptive radiation among section Sycomorus figs
Source: PLoS One. 2022 Apr 6;17(4):e0265339. doi: 10.1371/journal.pone.0265339 (PMC8985986; doi:10.1371/journal.pone.0265339)
Supplement: S1 Text — (DOCX) [file pone.0265339.s001.docx]

**Full typological description for *Bursaphelenchus suri* n. sp.**

Description in a traditional telegraphic style was not presented in main text because of the journal’s requirement to avoid overlapping of fixed phrases with previous species descriptions of typologically similar aphelenchoidid species. Therefore, the full description is given herein as supplementary material.

**Adult.** Relatively large and slender species of the genus. Body cylindrical, straight to ventrally weakly arcuate when killed by heat treatment. Cuticle of moderate thickness for genus, annulated, lateral field with four incisures, but the structure is vague, and internal lines are difficult to observe. Cephalic region distinctly offset, separated by a clear constriction. Lip separated into six (two dorsal, two subventral and two lateral) sectors, roundish rectangular to triangular in lateral view, *ca* twice as broad as high. A labial disc present at the anterior end, and its edge appears as two short projections in lateral view. Stylet with narrow lumen comprising a long cone of more than half of total stylet length and a shaft with clear and very well-developed and somewhat tear-drop-shaped basal swellings. Procorpus cylindrical, with clear procorpal tube at middle of cylinder, about two stylet lengths (about three metacorpal lengths) long, ending in a well-developed metacorpus (= median bulb). Metacorpus slightly elongate oval, muscular, and crescent-shaped metacorpal valve at middle of metacorpus. Dorsal pharyngeal gland orifice opening into lumen of metacorpus approximately midway between anterior end of metacorpus and metacorpal valve. Pharyngo-intestinal junction immediately posterior to metacorpus. Dorsal pharyngeal gland overlapping intestine dorsally, *ca* 7-10 metacorpal lengths long. Nerve ring surrounding pharyngeal glands and intestine at about one stylet length (about 1.5 metacorpal lengths) posterior to pharyngo-intestinal junction. Hemizonid distinct in permanently mounted material, about 1.5 stylet length (about 2 metacorpal lengths) posterior to metacorpus. Secretory-excretory pore located ventrally at immediately posterior or almost same level of hemizonid.

**Male.** Tail strongly curved ventrally when killed by heat. Body strongly ventrally arcuate in tail region. Gonad single, on the right of intestine, composed of testis and *vas deferens* from anterior. Anterior end of testis outstretched (8 out of 9 type specimens) or reflexed (1 out of 9). Spermatocytes arranged in multiple (3-5) rows in anterior 4/5 of testis, then developed sperm tightly packed in multiple rows in the posterior part of the testis. *Vas deferens* composed of relatively large and flattened cells, often harbouring sperm, but the margin between testis and *vas deferens* is not clear, probably because of poor condition of material. The posterior end of *vas deferens* fused with the posterior end of intestine to form a short and simple cloacal tube. Spicules typical for the *fungivorus* group of the genus, i.e., mitten-shaped in lateral view, with clear dorsal and ventral limbs, paired, separate. Dorsal limb conspicuous, ending ca one-fourth of spicule length from distal end of spicule. Anterior part of dorsal limb forming either a squared or blunt and triangular condylus, which is slightly dorsally truncate. Distal part of dorsal limb smoothly tapering to a pointed distal end. Rostrum distinctive, elongate triangular with pointed tip directed ventrally. Ventral limb conspicuous, thinner than dorsal limb, beginning immediately posterior to capitulum, straight, but slightly bent ventrally at one-third of distance from distal end. Thin, triangular, membrane-like cuticle connecting dorsal and ventral limbs. Cucullus absent. Gubernaculum or apophysis absent. Tail ventrally arcuate, smoothly tapering in anterior 2/3, and distal 1/3 narrowing to form a spike-like projection. Bursal flap conspicuous, covers the distal part from the level of P3, having an oval shape with a triangular projection at the posterior end. Six (three pairs) of genital papillae present, first subventral pair (P2) located immediately anterior to cloacal slit, second subventral pair (P3) located ca 65-70% of tail length from cloacal slit, third ventral pair (P4) immediately (ca 5 μm) posterior to P3. P2-P3 distance markedly (4-5 times) longer than P3-P4 distance. Ventral single precloacal papilla (P1) which is present in most other parasitaphelenchid nematodes not observed in light microscope, possibly vestigial.

**Female**. Reproductive tract located to the right of intestine, comprising ovary, oviduct, spermatheca, connection tissue, crustaformeria, uterus, vagina + vulva and post-uterine sac from anterior. Ovary single, anteriorly outstretched. Oocytes present in multiple (3-5) rows in anterior 2/3-4/5 of ovary, and well-developed oocytes arranged in a single row in the rest of ovary. Oviduct tube-like, connecting ovary and spermatheca. Spermatheca formed by distinctive thick tissue, not clearly forming an expansion or branch, i.e., slightly irregular oval in shape, sometimes filled with well-developed sperm. A cluster of small and rounded cells (regarded as ‘connective tissue’) present between spermatheca and crustaformeria. Crustaformeria not conspicuous, formed by relatively large and rounded cells. Uterus short, with thick wall. Vagina slightly inclined anteriorly, junction of uterus, post-uterine sac and vagina usually closed. A pair of three-celled structures usually found in the genus was not confirmed, possibly because of material condition, but this region was somewhat sclerotized. Vulva a simple slit lacking any flap apparatus in lateral view. Post-uterine sac long, 6.3-9.7 vulval body diam. long, extending for almost half or more (47-73%) of vulva to anus distance, sometimes filled with sperm. Anus and rectum present, seemingly functional. Tail slender, 5.6-10.7 anal body diam. long, straight or weakly recurved ventrally when killed by heat, cylindrical, forming elongate conoid, smoothly tapering to pointed terminus.
